# The community structure and microbial linkage of rumen protozoa and methanogens in response to the addition of tea seed saponins in the diet of beef cattle

**DOI:** 10.1186/s40104-020-00491-w

**Published:** 2020-08-12

**Authors:** Cui Tan, Carlos A. Ramírez-Restrepo, Ali Mujtaba Shah, Rui Hu, Matt Bell, Zhisheng Wang, Chris McSweeney

**Affiliations:** 1 Institute of Animal Nutrition, Sichuan Agricultural University, Chengdu, 611130 Sichuan China; 2 Commonwealth Scientific and Industrial Research Organisation, CSIRO Agriculture and Food, Australian Tropical Sciences and Innovation Precinct, James Cook University, Townsville, QLD 4811 Australia; 3 Present address: CR Eco-efficient Agriculture Consultancy (CREAC), 46 Bilbao Place, Bushland Beach, QLD 4818 Australia; 4 Department of Livestock Production, Shaheed Benazir Bhutto University of Veterinary and Animal Sciences, Sakrand, Sindh 67210 Pakistan; 5 “Low Carbon Breeding Cattle and Safety Production”, University Key Laboratory of Sichuan Province, Ya’an, 625014 Sichuan China; 6 School of Biosciences, University of Nottingham, Sutton Bonington, Loughborough, LE12 5RD UK; 7 CSIRO Agriculture, Queensland BioScience Precinct, St Lucia, Brisbane, QLD 4067 Australia

**Keywords:** Beef steers, Methane, Methanogen, Protozoa, Tea seed saponin

## Abstract

**Background:**

This study investigated changes in rumen protozoal and methanogenic communities, along with the correlations among microbial taxa and methane (CH_4_) production of six Belmont Red Composite beef steers fed tea seed saponins (TSS). Animals were fed in three consecutive feeding periods, a high-grain basal diet for 14 d (BD period) then a period of progressive addition of TSS to the basal diet up to 30 g/d for 20 d (TSS period), followed by the basal diet for 13 d without TSS (BDP post-control period).

**Results:**

The study found that TSS supplementation decreased the amount of the protozoal genus *Entodinium* and increased *Polyplastron* and *Eudiplodinium* genera. During BDP period, the protozoa community of steers did not return to the protozoal profiles observed in BD period, with higher proportions of *Metadinium* and *Eudiplodinium* and lower *Isotricha*. The addition of TSS was found to change the structure of methanogen community at the sub-genus level by decreasing the abundance of methanogens in the SGMT clade and increasing the abundance of methanogens in the RO clade. The correlation analysis indicated that the abundance of SGMT clade methanogens were positively correlated with *Isotricha*, and *Isotricha* genus and SGMT clade methanogens were positively correlated with CH_4_ production. While RO clade were positively correlated with the proportion of *Metadinium* genus, which was negatively correlated with CH_4_ emission.

**Conclusions:**

These results suggest that different genera of rumen protozoa ciliates appear to be selectively inhibited by TSS, and the change in methanogen community at the subgenus level may be due to the mutualistic relationships between methanogens and rumen ciliates.

## Background

Specific ciliate protozoa colonize the rumen ecosystem of ruminant animals and contribute 40% ~ 50% of the rumen microbial biomass, which is almost equal to that of rumen bacteria [1]. Rumen protozoa play an important role in feed digestion and homeostasis of the rumen ecosystem due to the predator-prey relationship and the symbiotic relationship between rumen ciliates and prokaryotic cells [2]. Studies using electron microscopy and sequencing revealed the presence of methanogens inside and outside of rumen ciliates [3–5]. And the higher methanogens/bacteria ratio observed within the protozoa fractions suggested that anaerobic ciliates contain symbiotic methanogens, which strengthened the association between rumen ciliates and methanogens [6]. The strong functional association between rumen protozoa and methanogens via interspecies hydrogen transfer contributes to overall methane (CH_4_) production (9% ~ 37%) in the rumen as demonstrated by defaunation or elimination of certain protozoa [7]. Thus, there is increasing evidence about the relationship between protozoa and methanogenic archaea, and their important role in rumen (CH_4_) formation [7–9].

Many rumen protozoa contain an organelle (hydrogenosome) which produces hydrogen that attracts methanogens as endosymbionts [10, 11]. Hydrogenosomes have been identified in species of *Epidinium*, *Isotricha* and *Dasytricha*, but they were not found in *Entodinium caudatum* and *Diploplastron affine* [12]. Tymensen et al. [4] reported that *Methanobrevibacter* and *Methanomicrobium* genera are the major groups of methanogen in the free-living (FL) and protozoa-associated methanogen (PAM) fractions, but their population structure differed between FL and PAM pools. In addition, the populations of PAM were more variable than FL methanogens [5]. It has been found that rumen methanogens closely related to *M. smithii* were primarily associated with the vestibuliferid protozoa (*Isotricha* and *Dasytricha*) [13]. While *Polyplastron multivesiculatum* harbored a large number of intracellular bacteria, no methanogen was observed [3]. Methanogens also associate with protozoa as ectosymbionts which may be a more stable population compared with the internal dwelling methanogens [11]. Due to the apparent variability in association between methanogens and different protozoal taxa, it is hypothesized that some protozoa species may have a greater effect on rumen methanogenesis and CH_4_ production than others.

Some studies have shown that variation in CH_4_ emissions are not necessarily due to the changes in methanogen cell density, but rather the community structure of the methanogens [14, 15]. Phytochemicals such as saponins, tannins and essential oils, have been used to manipulate rumen microbial populations and reduce CH_4_ emissions from ruminants [16]. Tea seed (*Camellia sinensis* L.) saponins (TSS) were reported to reduce methane production by an inhibitory effect on ciliate protozoa [17–19]. Tannins, essential oils, and linseed oil are also inhibitory to protozoa, but species are impacted differently [20–22]. Our previous study in beef cattle supplemented with TSS showed reduction in CH_4_ emissions were associated with changes in methanogen population structure, while total protozoal numbers remained unchanged [23]. It remains to be determined if there was an alteration of rumen ciliate composition in response to supplemental TSS, which further modified the methanogens composition and CH_4_ emissions. The recent development of sequencing primers that can discriminate members of the rumen protozoal populations may provide an opportunity to characterize changes in the relative abundance of different protozoal taxa that previously could only be determined by microscopy [24].

Therefore to gain greater insight into the community structure of ruminal ciliate protozoa and their relationship to methanogen diversity and CH_4_ production, we employed next-generation sequencing techniques to investigate these populations in the rumen of beef steers fed a high grain-based diet supplemented with TSS.

## Methods

### Animals, diet and experimental design

Care of animals and experimental procedures were approved by the CSIRO Animal Ethic Committee protocol No A12/2012. This research is part of a larger study that has been previously reported and see Ramirez-Restrepo et al. [23] for more details. Briefly, six Belmont Red Composite [Africander (African Sanga) × Brahman (*Bos indicus*) × Hereford-Shorthorn (3/4 *B. taurus*)] rumen-cannulated steers [327 ± 17.1 kg, initial body weight] were used in a repeated measures design.

After free grazing, the steers were individually allowed to adjust to a conventional-finishing feedlot basal diet (BD) fed ad libitum in two equal parts at 09:00 and 16:30 for 14 d (control BD period). This was followed by supplementing the BD in the morning feeding with 6, 10, 15, 20, 25 and 30 g/d of TSS light-yellow color powder dissolved in ~ 250 mL cold water during 5, 2, 4, 3, 2 and 4 d, respectively (TSS period). After that, using only the BD, steers were fed for other 13 days (post-control period; BDP). The basal diet consisted of Rhodes grass (*Chloris gayana*; 0.15) and a sorghum high-grain mixture (0.85; Coleman Stock Feeds Pty Ltd., Charters Towers, Australia; Additional file 1: Table S1). The TSS per kg of commercial product (Zhejiang Oriental Tea Technology Co., Ltd., Changshan, China) included 570 g triterpenoid saponin, 330 g crude fiber, 54 g crude protein, 50 g ash, and 1 g water-insoluble matter [25]. Water was available ad libitum and steers were allowed to exercise daily in the cattle yard before the morning feeding to reduce stress, record experimental data and facilitate pen cleaning.

Methane emissions from all steers were measured in open-circuit respiration chambers for each period, and the results of the six cattle in our previous paper [23] showed that the average CH_4_ production in the BDP period (14.2 g/kg DMI) was significantly lower than the CH_4_ production in the BD period (18.0 g/kg DMI) and TSS period (18.7 g/kg DMI) respectively (*P* < 0.05). The lower CH_4_ emissions in BDP period could be due to the reestablishment of a microbial community that had lower CH_4_ producing potential than the controls.

### Analysing protozoal and methanogenic communities

Rumen samples from each rumen-cannulated steer at the end of each feeding period were collected 2 h after the morning feeding. Raft and liquid components of the rumen content obtained from the dorsal and ventral sacks and were squeezed through two layers of cheese cloth. The fluid was immediately transferred to sterile containers placed on dry ice, and stored at − 80 °C for later DNA extraction.

Genomic DNA was extracted from 2 mL rumen fluid using the standard cetyl-trimethyl ammonium bromide (CTAB) method with some modifications to obtain higher concentrations and better purity of gDNA extracts from the rumen fluid [26]. The procedure used for DNA extraction is described in detail in our previous study [23] using bead beating in CTAB lysis buffer followed by phenol–chloroform separation. DNA extracts were dissolved in a 200 μL buffer EB, and the quality and quantity of the extracted DNA were determined by UV spectrophotometric analysis, using a NanoDrop ND-1000 Spectrophotometer (Nyxor Biotech, Paris, France).

The V3–V4 region of the protozoan 18S rRNA gene was PCR amplified using PSSU316F-GIC758R [24] barcoded primers set, and the V3–V4 region of the methanogen 16S rRNA gene was amplified using 915F-1386R [27] barcoded primers set. The two-step PCR amplification procedure with the barcoded primers (Additional file 2: Table S2) were used to obtain the PCR amplicons for paired-end sequencing on an Illumina MiSeq platform. The first-step PCR amplification setup consisted of 10 μL 5× Phusion HF buffer (including MgCl_2_), 1 μL dNTP (10 mmol/L), 1 μL inner primer (10 μmol/L), 20–50 ng of DNA template 1 U of Phusion High Fidelity DNA Polymerase (New England Biolabs, Beijing, China) and ddH_2_O up to 50 μL. Amplification was initiated with denaturation for 2 min at 94 °C, followed by 30 cycles of 94 °C for 30 s, annealing at 55 °C for protozoa or 56 °C for methanogen amplifying for 1 min and 30 s and extension at 72 °C for 30 s, with a final elongation for 2 min. After purification of PCR product, the cleaned PCR fragments were used in the second step PCR amplification. The 2^nd^-step PCR setup consisted of 8 μL HF buffer, 1 μL dNTP, 1 μL outer primer, 5 μL cleaned PCR product, 0.8 U of DNA Polymerase and ddH_2_O up to 40 μL. PCR cycles were as follows: 1 cycle at 94 °C for 2 min; 8 cycles at 94 °C for 30 s, 56 °C for 1.5 min and 72 °C for 30 s; and a final extension step at 72 °C for 2 min.

The purified barcoded amplicons were pooled in equimolar quantity to generate the Illumina library and quantified on Qubit 3.0 Fluorometer (Invitrogen, Waltham, USA). Sequencing was conducted on the Illumina multiplex 2 × 300-bp paired-end MiSeq-platform in TINY gene biotechnology Co. Ltd. (Shanghai, China). The sequencing procedure was followed as per Kozich et al. [28]. The raw sequencing data were submitted to the NCBI Sequence Read Archive (SRA), which are available from NCBI under BioProject accession number PRJNA574788 (https://www.ncbi.nlm.nih.gov/bioproject/ PRJNA574788) for both protozoal (*n* = 18) and methanogenic (*n* = 18) community datasets.

The paired-end reads were assembled by using Mothur software package [29], and then trimmed and quality-filtered in QIIME using default settings [30]. Briefly, Paired-end reads with ambiguous bases (*n* > 0), homologous regions (*n* ≥ 8), mismatches in the primer (*n* > 0), nontarget areas and errors in barcodes (*n* > 0) were culled, as well as sequences less than 200 bp, longer than 580 bp. Subsequently, chimeric sequences were removed by UCHIME [31]. And the quality-filtered sequences were clustered into operational taxonomic units (OTUs) for species classification with 96% similarity for ciliate protozoa [24]. An 18S rRNA gene sequence reference, providing the alignment for candidate sequences of ciliate protozoa, contained 131 sequences (≥1,500 bp in length) belonging to the gastrointestinal tract trichostome (subclass Trichostomatia) ciliates and downloaded from NCBI. The sequences of methanogen dataset were assigned to OTUs at a 3% species-level cutoff [32], with the RDP (Ribosomal Database Project) database as the reference. For OTUs grouping, only one or two reads were removed before taxonomic assignment. A good depth of coverage, community diversity indicators, the Shannon-Wiener index, Simpson’s index and community richness estimates, such as the Chao 1 index, were also calculated using Mothur and self-compiled employing program in BioLinker. Phylogenetic tree was constructed to visualize the phylogeny of dominant methanogen OTUs, representing sequences across all samples, using the neighbor-joining method in the MEGA6 [33].

### Statistical analyses

All statistical analysis was performed using the R Package (version 3.5.1). Normality test of all the data was analyzed first. The normally distributed data, included OTU richness and diversity indices, were analyzed by using one-way analysis of variance (ANOVA) followed by the application of Tukey test for multiple comparison. The non-normally distributed data, taxonomic richness, were analyzed by using Kruskal-Wallis test to assess overall significant differences among the groups, followed by the post hoc Wilcoxon rank sum test to determine which paired groups differed from each other. Unless otherwise noted, the mean and pooled standard error of the mean (SEM) are reported. Spearman correlation analysis was used to assess microbial community interactions between protozoa and methanogen, as well as the relationship between microbial taxa abundance and CH_4_. Correlation matrix and graphics were generated using the online resource (https://www.omicshare.com/). *P*-values of < 0.05 for the overall significant differences and *P*-values of < 0.05 after FDR correction using the Benjamini–Hochberg procedure for the multiple comparison were considered significant.

## Results

### Effect on rumen protozoa communities

A total of 457,733 high-quality sequences mapped to protozoan 18S rRNA references generated 1,473 representative OTUs at 96% similarity, for an average of 870 OTUs per sample (range 593–1208). TSS supplementation reduced the number of observed protozoa OTUs and the species richness metrics, Chao 1 and ACE (FDR-*P* < 0.05; Table 1). This indicates a reduction in protozoal species richness for beef steers fed on a diet containing TSS. However, the alpha diversity indices of rumen protozoa, Shannon-Wiener and Simpson’s indices, were not influenced by TSS supplementation (*P* > 0.05, Table 1), so the reduction of species richness and similarity of alpha diversity indices with TSS indicated a shift in the evenness of protozoa taxa due to TSS. Good’s Coverage index showed good overall sampling and sequencing, ranging between 96.9% and 98.5% for rumen protozoa.

**Table 1:** Effect of tea seed saponins (TSS) supplementation on OTU richness and alpha diversity of ciliate protozoa at 96% similarity

Treatment	BD	TSS	BDP	SEM	ANOVA_*P*
Observed OTUs	999^a^	771^b^	840^ab^	37	0.354
Shannon-Wiener	3.04	3.32	2.98	0.08	0.173
Simpson’s	0.13	0.10	0.14	0.01	0.458
Chao 1	2,699^a^	1,805^b^	2,162^ab^	125	0.005
Ace	4,223^a^	2,599^b^	3,186^ab^	226	0.004
Good’s coverage, %	97.78	98.02	97.68	0.13	0.613

Within the same row, values with a different letter are significantly different (FDR-*P* < 0.05)

ANOVA_*P* values were the overall significant differences analyzed using one-way analysis of variance for the normally distributed data

BD Basal Diet, *BDP* Post-control period, *SEM* Standard error of the mean

Across all samples, taxonomic classification based on the observed OTUs at the family and genus level resulted in the identification of 3 families and 10 genera of protozoa. The families Ophryoscolecidae and Isotrichidae accounted for 85.9% and 8.7% of the sequences, respectively (Additional file 3: Figure S1A). *Entodinium* (44.1%), *Metadinium* (10.5%), *Eudiplodinium* (5.9%) and *Polyplastron* (4.7%) were the dominant genera identified in the family Ophryoscolecidae. Another two genera in the family Isotrichidae, *Isotricha* (8.0%) and *Dasytricha* (0.7%) were also identified in this study (Additional file 3: Figure S1B). And the community composition of rumen protozoa at the order, family and genus level among three periods are shown in Additional file 3: Figure S2. At the order and family level, less relative abundance of Entodiniomorphida and Ophryoscolecidae were observed in TSS supplementation steers (FDR-*P* < 0.05) compared with BDP period, while the abundance of Vestibuliferida order and Isotrichidae family in BDP period were much lower than that of BD and TSS periods (FDR-*P* < 0.05, Table 2). At the genus level, TSS supplementation significantly reduced the abundance of *Entodinium* (FDR-*P* < 0.05), while significantly increased the abundance of the *Polyplastron* genus (FDR-*P* < 0.05, Table 2). Meanwhile, the abundance of *Eudiplodinium* in BDP period was higher than the BD period (FDR-*P* < 0.05). As for the *Metadinium* genus, its abundance in BDP period was higher than that in other two periods (FDR-*P* < 0.05), but the abundance of *Isotricha* in BDP period was lower (FDR-*P* < 0.05, Table 2).

**Table 2:** Effect of tea seed saponins (TSS) on the protozoal communities (% of total sequences)

Assigned taxa	BD	TSS	BDP	SEM	KW_*P*
O-Entodiniomorphida	83.94^ab^	70.50^b^	94.96^a^	3.51	0.007
F-Ophryoscolecidae	83.73^ab^	70.25^b^	94.76^a^	3.54	0.007
G-*Entodinium*	52.44^a^	29.45^b^	39.72^ab^	4.11	0.009
G-*Metadinium*	8.32^b^	6.67^b^	17.43^a^	1.97	0.048
G-*Polyplastron*	1.72^b^	9.10^a^	6.22^ab^	1.13	0.017
G-*Eudiplodinium*	2.46^b^	7.62^ab^	9.71^a^	1.14	0.030
G-*Ostracodinium*	1.00	2.81	2.08	0.60	0.806
G-*Ophryoscolex*	0.66	4.18	0.98	0.85	0.463
O-Vestibuliferida	12.78^a^	13.74^a^	2.99^b^	2.03	0.007
F-Isotrichidae	12.78^a^	13.73^a^	2.99^b^	2.03	0.008
G-*Isotricha*	11.82^a^	12.74^a^	2.83^b^	1.91	0.008
G-*Dasytricha*	0.95	0.97	0.41	0.16	0.211
O-Unclassified	3.32^b^	15.86^a^	2.07^b^	2.25	0.011

Values within the same row with different letters are significant different (FDR-*P* < 0.05)

KW_*P* values were the overall significant differences analyzed using Kruskal-Wallis test for the non-normally distributed data

*BD* Basal Diet, *BDP* Post-control period, *SEM* Standard error of the mean. O Order, F Family, G Genus

### Effect on rumen methanogenic communities

A total of 370,065 high-quality sequences were mapped to methanogen 16S rRNA references and generated 1,441 representative OTUs at 97% similarity, for an average of 459 OTUs per sample. The number of observed methanogen OTUs in the rumen of beef steers fed on a basal diet was not affected by TSS supplementation, while higher ACE index for rumen methanogens was observed in TSS supplementation steers compared with BDP period (FDR-*P* = 0.08, Table 3). However, the alpha diversity indices of rumen methanogen, Shannon-Wiener and Simpson’s indices, were not affected by TSS supplementation (Table 3).

**Table 3:** Effect of tea seed saponins (TSS) supplementation on OTU richness and alpha diversity of methanogen at 97% similarity

Treatment	BD	TSS	BDP	SEM	ANOVA_*P*
Observed OTUs	447	490	443	12	0.167
Shannon-Wiener	2.13	2.40	2.45	0.10	0.391
Simpson’s	0.36	0.31	0.31	0.02	0.691
Chao 1	1251	1335	1175	48	0.421
Ace	1761	2073	1672	78	0.083
Good’s coverage, %	98.28	97.05	96.81	0.23	0.211

Within the same row, values with different letters were significantly different (FDR-*P* < 0.05)

ANOVA_*P* values were the overall significant differences analyzed using one-way analysis of variance for the normally distributed data

*BD* Basal Diet, *BDP* Post-control period, *SEM* Standard error of the mean

Across all samples, the methanogen OTUs annotated to 2 families and 5 genera (Table 4). Methanobacteriaceae and *Methanobrevibacter* spp. were the predominant family and genera of methanogen in the rumen of steers during the three periods, accounting for more than 98% of the sequences. The abundance of methanogens at the family and genus levels were not significantly affected by TSS supplementation, while the abundance of *Methanosphaera* in BDP steers tended to be higher than in the BD period (FDR-*P* = 0.08; Table 4).

**Table 4:** Effect of tea seed saponins (TSS) on the methanogen communities (% of total sequences)

Assigned taxa	BD	TSS	BDP	SEM	KW_*P*
O-Methanobacteriales	99.42	98.55	99.55	0.22	0.440
F-Methanobacteriaceae	99.42	98.55	99.55	0.22	0.440
G-*Methanobrevibacter*	99.06	98.09	98.94	0.22	0.338
G-*Methanosphaera*	0.04^b^	0.10^ab^	0.14^a^	0.02	0.010
G-*Methanosarcina*	0.01	0.01	0.01	0.00	0.892
O-Thermoplasmatales	0.51	1.29	0.37	0.20	0.446
G-*Candidatus Methanomethylophilus*	0.51	1.29	0.37	0.20	0.446
O-Methanomicrobiales	0.03	0.06	0.03	0.01	0.854
F-Methanomicrobiaceae	0.03	0.06	0.03	0.01	0.854
G-*Methanomicrobium*	0.03	0.05	0.03	0.01	0.720

Within the same row, values with a different letter were significantly different (FDR-*P* < 0.05)

KW_*P* values were the overall significant differences analyzed using Kruskal-Wallis test for the non-normally distributed data

*BD* Basal Diet, *BDP* Post-control period, *SEM* Standard error of the mean. O Order, F Family, G Genus

### Effect on the dominant methanogen OTUs

**Fig. 1:**
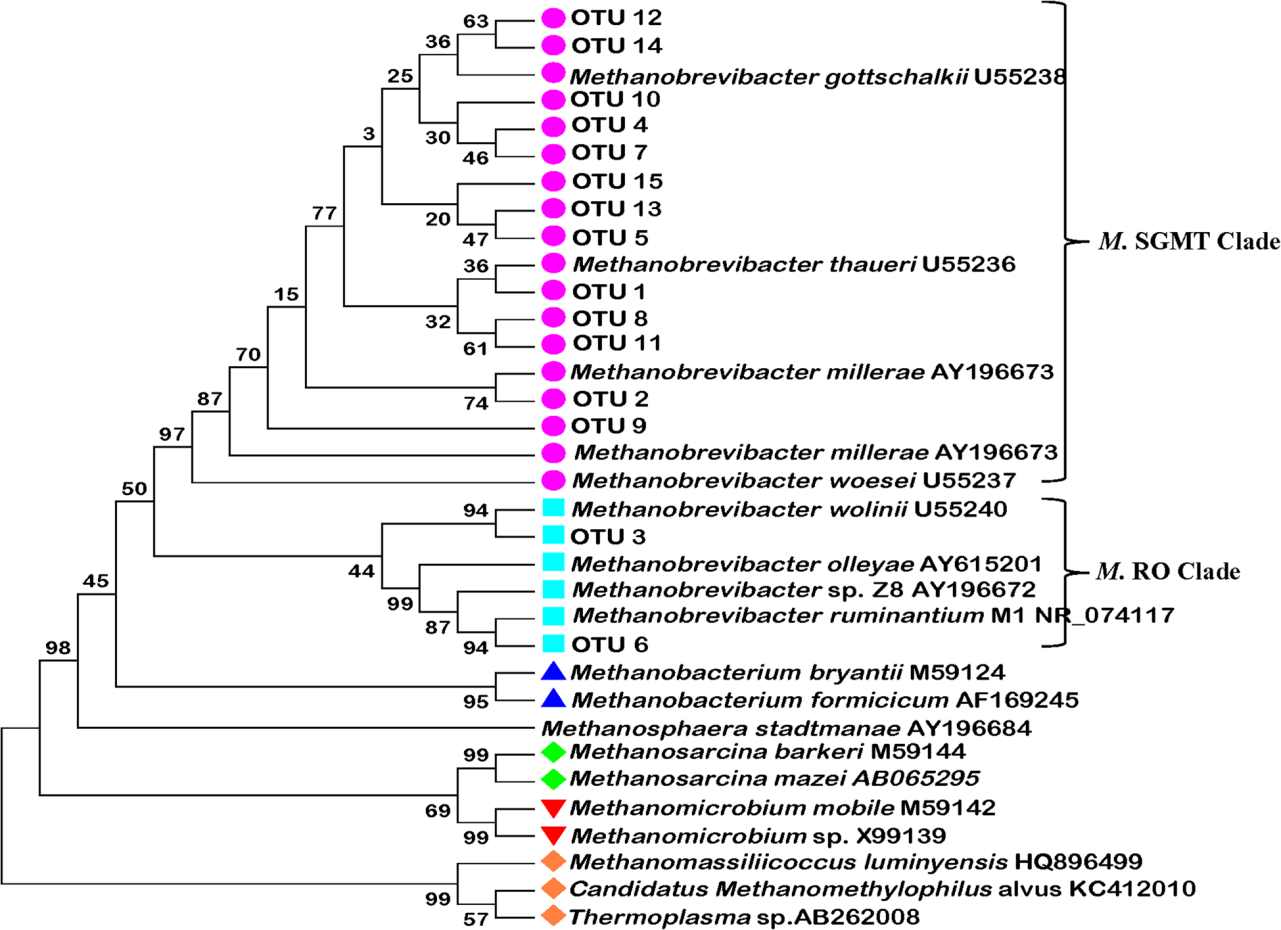
Phylogenetic relationships between the 15 dominant methanogenic OTUs of all steers and *Methanobrevibacter* spp. The sequences of 15 dominant methanogenic OTUs obtained from 16S rRNA illumina library prepared from the community DNA of rumen of Belmont Red Composite steers, and the reference sequences of the *Methanobrevibacter* genus obtained from GenBank. The phylogenetic tree was constructed using the Neighbor-Joining method with the Kimura two-parameter model

There were 15 dominant methanogen OTUs, averaging greater than 1% of total methanogen sequences across all samples, were used to construct the phylogenetic tree (Fig. 1). The results showed that 86.7% (13 OTUs) of the methanogen OTUs were located in the SGMT clade of the genus *Methanobrevibacter* (*M. smithii*, *M. gottschalkii*, *M. millerae* and *M. thauerii*), only 13.3% (2 OTUs) were located in the RO cluster of the genus *Methanobrevibacter* (*M. ruminentium* and *M. olleyae*).

The two predominant OTUs, OTU 1 and OTU 2, both representing approximately 30% of all sequences, were located in the SGMT clade of the *Methanobrevibacter* genus. The relative abundances of the 15 dominant methanogen OTUs were compared among the three feeding periods (Table 5). Compared with BD period, TSS supplementation significantly reduced the relative abundance of OTU 4 and OTU 7 (FDR-*P* < 0.05), while increased the proportion of OTU 5 and OTU 6 (FDR-*P* < 0.05). However, the abundance of SGMT clade and OTU 1 in BDP period was much lower than that in the BD period (FDR-*P* < 0.05), while the proportion of RO clade of the *Methanobrevibacter* genus and OTU 6 in BDP period was higher than BD period (FDR-*P* < 0.05).

**Table 5:** Effect of tea seed saponins (TSS) on the abundance of dominant methanogen clade/OTUs

Methanogen clade/OTUs	BD	TSS	BDP	SEM	KW_*P*
SGMT clade	74.95^a^	62.15^ab^	42.14^b^	4.16	0.005
OTU 1	20.97^a^	32.23^a^	9.14^b^	2.78	0.009
OTU 2	14.75	9.25	8.05	1.09	0.172
OTU 4	10.78^a^	0.88^b^	4.68^ab^	1.21	0.017
OTU 5	2.06^b^	13.99^a^	4.07^b^	1.45	0.002
OTU 7	10.91^a^	0.12^b^	2.18^ab^	1.40	0.001
Other 8 OTUs	15.49^a^	5.68^b^	14.01^ab^	1.80	0.027
RO clade	11.72^b^	17.99^ab^	35.52^a^	3.07	0.009
OTU 3	7.63	9.70	20.87	1.77	0.164
OTU 6	4.09^b^	8.29^a^	14.65^a^	1.66	0.002

SGMT clade: *M. smithii, M. gottschalkii, M. millerae* and *M. thauerii*, RO clade: *M. ruminentium* and *M. olleyae*

Other 8 OTUs are the OTU 8, OTU 9, OTU 10, OTU 11, OTU 12, OTU 13, OTU 14, OTU 15

In the same row, values with different letter mean significant difference (FDR-*P* < 0.05)

*BD* Basal Diet, *BDP* Post-control period, *SEM* Standard error of the mean

KW_*P* values were the overall significant differences analyzed using Kruskal-Wallis test for the non-normally distributed data

### Within- and between-domain associations among protozoa and methanogen

**Fig. 2:**
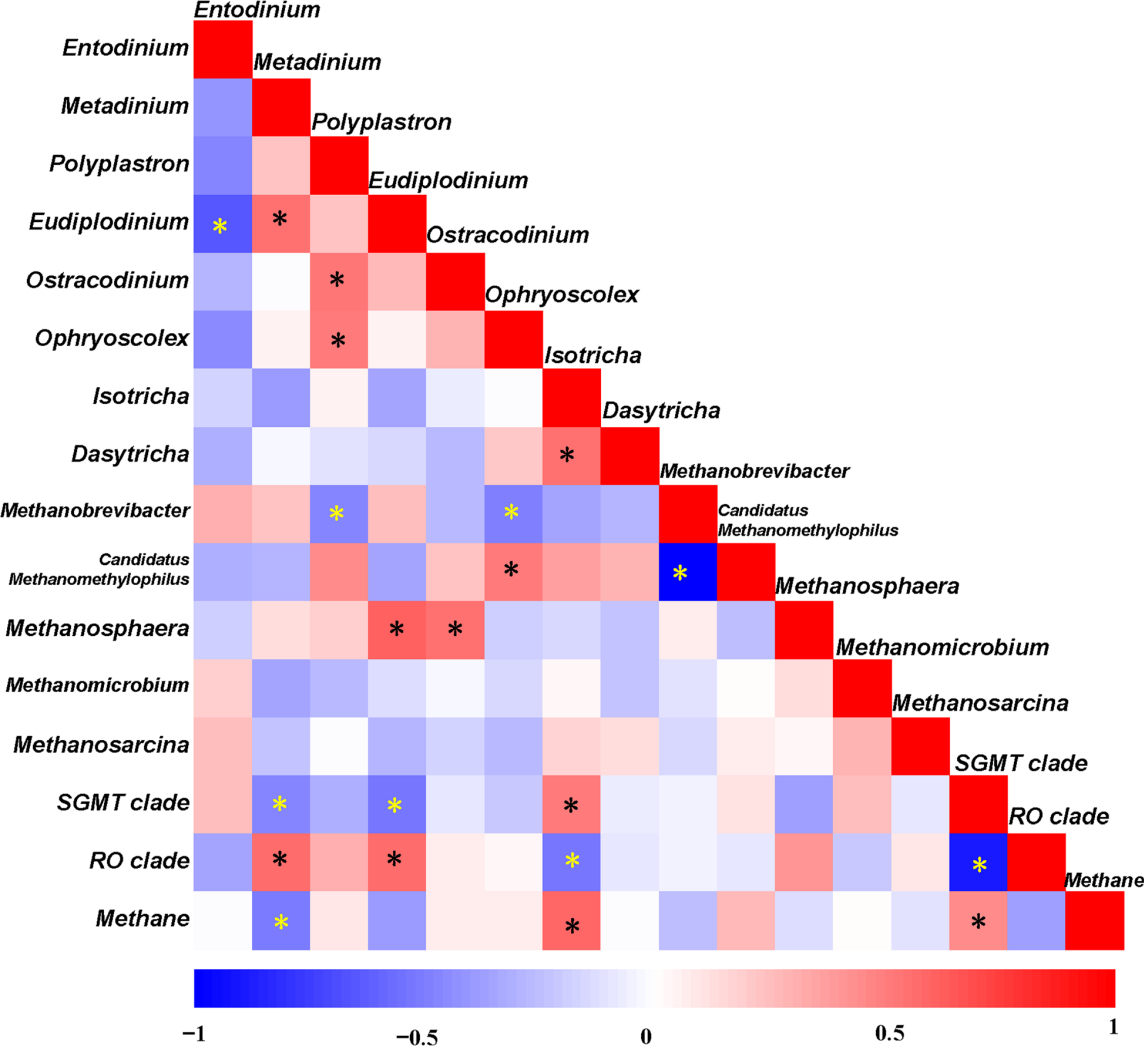
Correlation matrix between protozoal taxa with methanogens and methane. Positive correlations are shown in red and negative correlations in blue. Color intensity represents *r*-values of correlation, ∗indicates *P* < 0.05. The scale of the colors is denoted as follows: the more positive the correlation (closer to 1), the darker the shade of red; the more negative the correlation (closer to − 1), the darker the shade of blue. ∗indicates *P* < 0.05

Pearson’s correlations indicated significant associations among protozoa genera, and between protozoa genera and methanogen taxa. Within ciliate protozoa, positive correlations were observed between the abundance of *Metadinium* and *Eudiplodinium* (*r* = 0.55; *P* = 0.02), between *Isotricha* and *Dasytricha* (*r* = 0.55; *P* = 0.02), as well as between *Polyplastron* genus with *Ostracodinium* and *Ophryoscolex* (*r* = 0.54 and 0.51, respectively; *P* < 0.05), and strong negative correlation was noticed between *Entodinium* and *Eudiplodinium* (*r* = − 0.66; *P* = 0.01; Fig. 2). In relation to protozoa-methanogen interactions, negative correlations were observed between the *Methanobrevibacter* genus with *Polyplastron* and *Ophryoscolex* (*r* = − 0.48 and − 0.49, respectively; *P* < 0.05). While positive correlations occurred between the *Methanosphaera* genus with *Eudiplodinium* and *Ostracodinium* (*r* = 0.62 and 0.56, respectively; *P* < 0.05), as well as between the *Methanomethylovorans* genus with *Polyplastron* and *Ophryoscolex* (*r* = 0.48 and 0.54, respectively; *P* < 0.05; Fig. 2). More significant correlations between the abundance of protozoal genera and the two clades of the genus *Methanobrevibacter*. SGMT clade was positively correlated with *Isotricha* (*r* = 0.51; *P* = 0.03), and negatively with *Eudiplodinium* and *Eudiplodinium* (*r* = − 0.49 and 0.51, respectively; *P* = 0.03), the RO clade was positively correlated with *Metadinium* and *Eudiplodinium* (*r* = 0.57 and 0.56, respectively; *P* < 0.05), and negatively with *Isotricha* (*r* = − 0.51; *P* = 0.01; Fig. 2).

### Correlations between microbial taxa with rumen CH_4_ emission

The yield of CH_4_ per unit of DMI (dry matter intake) was positively correlated with the relative abundance of *Isotricha* genus (*r* = 0.59, respectively; *P* < 0.05), and negatively correlated with the genus *Metadinium* (*r* = − 0.50; *P* < 0.05). As for methanogens, only SGMT clade of the genus *Methanobrevibacter* was positively correlated with CH_4_ production (*r* = 0.46; *P* = 0.05), while no significant correlation between CH_4_ emissions and methanogen abundance at the genus and family level were observed (Fig. 2).

## Discussion

Rumen microbial community is shaped by complex relationships between bacteria, ciliate protozoa, fungi and archaea. Previous studies have shown that TTS as a feed additive can potentially mitigate CH_4_ emissions from ruminants through their inhibitory effect on protozoa, which subsequently may alter the abundance/diversity of methanogens [18, 34]. In the present study, TSS supplementation reduced the protozoal diversity metrics, but diversity still remained high throughout the study. We speculate that the high value diversity metrics is probably linked with the high-concentrate diet (concentrate to forage, 75:25) used, which can promote the growth of rumen ciliates [35, 36]. Furthermore, the high 18S rRNA gene copies number for large entodiniomorphids protozoa can partially explain the high diversity of rumen protozoa in this study [37]. In this study, the rumen ciliates were taxonomically assigned to 3 protozoan families and 10 genera. The genus *Entodinium* spp. was the predominant genus in the most dominant family, Ophryoscolecidae, comprising up to 85% of the total ciliate population, which is in agreement with other studies with cattle and sheep [27, 38]. In the present study, the TSS supplementation in a grain-based diet modified the community composition of rumen ciliate protozoa. The relative abundance of the family Ophryoscolecidae and the *Entodinium* genus in particular were reduced by TSS, however, concurrently there was an increase in the relative proportion of *Polyplastron* and *Eudiplodinium* genera, which demonstrated that there was a significant selective inhibitory effect of TSS on the ciliate protozoa community at the family and genus level. This selective inhibitory effect of TSS on rumen protozoa could be due to the physical or chemical differences in the cilia, the pellicle or metabolic activity of rumen ciliates. The concentration and composition of ciliate protozoa in the rumen can be influenced by diet type, pH, supplements, concentrate and roughage level [35, 39]. The most consistent effect of tea saponin supplementation is a decrease in the abundance of rumen protozoa in vitro [40], as well as in the rumen of goats and sheep [17, 18].

Most Ophryoscolecidae ciliates seem to be able to harbor both intracellular and extracellular methanogens, whereas only endosymbiont methanogens have been observed with the Isotrichidae ciliates [41]. In addition, previous studies have reported that *Entodinium* spp. and *Isotricha* spp. contain intracellular methanogens, whereas *Polyplastron multivesiculatum* harbored mainly intracellular bacteria [42]. In the present study, after the withdrawal of TSS supplementation, the ciliate community structure was also dissimilar with a higher proportion of *Metadinium*, *Polyplastron* and *Eudiplodinium* and lower *Isotricha*, which may also affect other rumen microbial communities due to the predator-prey relationship and the symbiotic relationship between rumen ciliates and prokaryotes (rumen bacteria and methanogens) [43]. The CH_4_ production in the rumen is significantly influenced by the protozoa population due to inter-species transfer of hydrogen from the protozoa to methanogens [44]. It has also been suggested that the number and activity of endosymbiotic population of methanogens are greater than the ectosymbionts attached to the surface of protozoa [45]. In most cases, the variation in CH_4_ emissions, caused by the addition of plant secondary compounds or microbial modifiers, could not be attributed to differences in methanogen cell density, but rather the composition of the methanogen community [46]. These results suggest that the shift of the ciliate community composition may result in dissimilarity in methanogen community that could vary in their potential for CH_4_ production.

In this study, the Methanobacteriaceae and *Methanobrevibacter* spp. were the predominant family and genus of methanogen in the rumen of steers throughout the trial, which is consistent with the previous findings [47, 48]. We suggest that this may be related to the high-concentrate diet used in our study. As Hook et al. [35] reported, approximately 99.3% of methanogen clones were assigned to the genus *Methanobrevibacter* in the rumen of cattles fed 65% concentrate diet. Furthermore, Hook et al. [35] and Franzolin [36] found that a high-concentrate diet promoted the abundance of protozoa. And other studies have found that the *Methanobrevibacter* was the predominant genus associated with protozoa [4, 5]. Therefore, the high density of rumen protozoa may contribute to the dominance of *Methanobrevibacter* in the rumen of catle fed the high-concentrate diet. St-Pierre et al. [46] observed that *Methanobrevibacter* related methanogen 16S rRNA gene sequences are mainly distributed between two large clades: SGMT clade, closely related to *M. smithii*, *M. gottschalkii*, *M. millerae* and *M. thaueri*, and RO clade, closely related to *M. ruminantium* and *M. olleyae*. The phylogenetic tree of the 15 dominant methanogen OTUs in this study showed that 86.7% methanogen OTUs, accounted for more than 70% of the sequences, and were located in the SGMT clade of the genus *Methanobrevibacter* with only 13.3% OTUs located in the RO clade. A re-examining report compared the sequence distribution between the SGMT clade and the RO clade and found that methanogens in the SGMT clade were distinctively more highly represented than the RO clade in impalas, wallabies, Holstein dairy cows and water buffaloes [44], which is consistent with the results of the present study. In the current study, the lower abundance of SGMT clade and higher proportion of RO clade were observed after the withdrawal of TSS supplementation, which was also associated with the lower proportion of *Isotricha* and higher *Metadinium*. What’s more, a positive correlation was observed between SGMT clade methanogens and *Isotricha*, as well as between the RO clade for *Metadinium*. Therefore, changes in the structure and composition of protozoan community should alter the structure of the methanogen community at the subgenus level.

The correlations between microbial taxa with CH_4_ emissions in the present study showed that CH_4_ production was positively correlated with *Isotricha*, but negatively correlated with *Metadinium*. This finding is supported by a previous study which reported that CH_4_ production and methanogen population in sheep inoculated with Isotrichidae ciliates were similar to the total protozoa of inoculated sheep [49]. The correlation analysis also showed that CH_4_ production was positively correlated with the abundance of SGMT clade methanogens. The results indicated that the shift in the methanogen community profile at the subgenus level were more effective in producing CH_4_, which could help explain the lower amount of CH_4_ emissions in the steers after withdrawing TTS from the diet, with a reduction in predominant methanogen species (SGMT clade methanogens) and increase in minor species (RO clade methanogens).

## Conclusions

Adding TSS in the basal diet of beef steers caused significant alterations in the rumen protozoa community structure at the family and genus level, as well as rumen methanogen community structure. After withdrawing TSS, the ciliate community of steers did not return to the same protozoa community prior to TSS supplementation, and the new structure of the rumen microbial community maybe shaped by complex relationship between ciliate protozoa and methanogenc archaea. A number of protozoa genera and methanogen species were found to be correlated with CH_4_ emissions. Therefore, we speculated that decreasing the abundance of protozoa belonging to the *Isotricha* genus, and increasing members of the *Metadinium* genus, are likely to reduce CH_4_ emissions.

## Supplementary information

**Additional file 1 Table S1.** Table S1 Composition and nutrient concentration of the basal diet.

**Additional file 2 Table S2.** The primers used in the construction of Illumina sequencing library.

**Additional file 3 Figure S1.** Relative abundance of ruminal protozoa genera of steers at the family level (A) and the genus level (B).

**Additional file 3 Figure S2.** The community composition of rumen protozoa at the order (A), family (B) and genus (C) level among three periods.

## Data Availability

All sequencing data are available in the NCBI Sequence Read Archive (SRA), under the bioproject number PRJNA574788.

## Abbreviations

TSS: Tea seed saponins
BD: Basal diet
BDP: Basal diet post
OTU: Operational taxonomic unit
SGMT: *M.****s****mithii*, *M.****g****ottschalkii*, *M.****m****illerae* and *M.****t****hauerii*
RO: *M.****r****uminentium* and *M.****o****lleyae*
