# Supplementary material for: The community structure and microbial linkage of rumen protozoa and methanogens in response to the addition of tea seed saponins in the diet of beef cattle
Source: J Anim Sci Biotechnol. 2020 Aug 12;11:80. doi: 10.1186/s40104-020-00491-w (PMC7422560; doi:10.1186/s40104-020-00491-w)
Supplement: Supplementary file 1 — Additional file 1 Table S1. Table S1 Composition and nutrient concentration of the basal diet. [file 40104_2020_491_MOESM1_ESM.docx]

**Table S1** Ingredients and nutrient concentration of the basal diet

|  | Commercial high-grain mixture^†^ | Rhodes grass hay | Offered mixed ration, g/kg |
| --- | --- | --- | --- |
| Ingredient, g/kg DM |  |  |  |
| Rhodes grass hay |  |  | 150 |
| Commercial high-grain mixture |  |  | 850 |
| Sorghum 10% crushed | 594 |  |  |
| Peanut shells | 110 |  |  |
| Forage hay | 90 |  |  |
| Concentrate | 60 |  |  |
| Sodium-Bentonite | 20 |  |  |
| Sodium Bicarbonate | 5 |  |  |
| Mildew inhibitor | 1 |  |  |
| Molasses 120 | 120 |  |  |
| Nutrient concentration, g/kg DM |  |  |  |
| DM | 877.0 | 896.0 | 879.9^ϯ^ |
| Crude protein | 128.9 | 79.0 | 121.4^ϯ^ |
| Crude fibre | 116.8 | 401.0 | 159.4^ϯ^ |
| Ether extract | 22.2 | 14.0 | 21.0^ϯ^ |
| Ca | 8.3 | 4.1 | 7.6^ϯ^ |
| P | 2.7 | 0.90 | 2.4^ϯ^ |
| Na | 4.7 | 3.1 | 4.5^ϯ^ |
| Metabolisable energy, MJ/kg DM | 10.2 | 8.0 | 8.8^ϯ^ |

^†^ Included S 1.0 g, Co 0.48 mg, Cu 0.10 mg, Fe 30.05 mg, I 0.48 mg, Mg 0.16 mg, Mo 0.018 mg, Zn 44.0 mg and vitamins A 2.28 mg, D3 38.30 µg and E 6.39 mg per kg of commercial product. DM= dry matter.

^Ϯ^ Value proportionally derived.
