# Supplementary material for: The community structure and microbial linkage of rumen protozoa and methanogens in response to the addition of tea seed saponins in the diet of beef cattle
Source: J Anim Sci Biotechnol. 2020 Aug 12;11:80. doi: 10.1186/s40104-020-00491-w (PMC7422560; doi:10.1186/s40104-020-00491-w)
Supplement: Supplementary file 2 — Additional file 2 Table S2. The primers used in the construction of Illumina sequencing library. [file 40104_2020_491_MOESM2_ESM.doc]

**Table S2** Sequence of primers used in the construction of Illumina library

| Polymerase chain reaction (PCR) stages | Primers | Oligonucleotide sequence (5’ - - 3’) |
| --- | --- | --- |
| First step | Inner primers |  |
| Protozoan specific primers | P-SSU-316F | TTCCCTACACGACGCTCTTCCGATCT**GCTTTCGWTGGTAGTGTATT** |
|  | GIC758R | GAGTTCCTTGGCACCCGAGAATTCCA**CAACTGTCTCTATKAAYCG** |
| Methanogenic specific primers | 915F | TTCCCTACACGACGCTCTTCCGATCT**AGGAATTGGCGGGGGAGCAC** |
|  | 1386R | GAGTTCCTTGGCACCCGAGAATTCCA**GCGGTGTGTGCAAGGAGC** |
| Second step | Outer primers |  |
|  | Forward | AATGATACGGCGACCACCGAGATCTACAC-NNNNNNNN-TCTTTCCCTACACGACGCTC |
|  | Reverse | CAAGCAGAAGACGGCATACGAGAT-NNNNNNNN-GTGACTGGAGTTCCTTGGCACCCGAGA |

Underlined bases represent overhang Illumina adapter sequences; bold-face bases represent the gene specific PCR primers;

dotted-underline bases represent sequencing primer sequences; and eight N indicate barcode sequences.
