# Supplementary figures and images for: The community structure and microbial linkage of rumen protozoa and methanogens in response to the addition of tea seed saponins in the diet of beef cattle
Source: J Anim Sci Biotechnol. 2020 Aug 12;11:80. doi: 10.1186/s40104-020-00491-w (PMC7422560; doi:10.1186/s40104-020-00491-w)

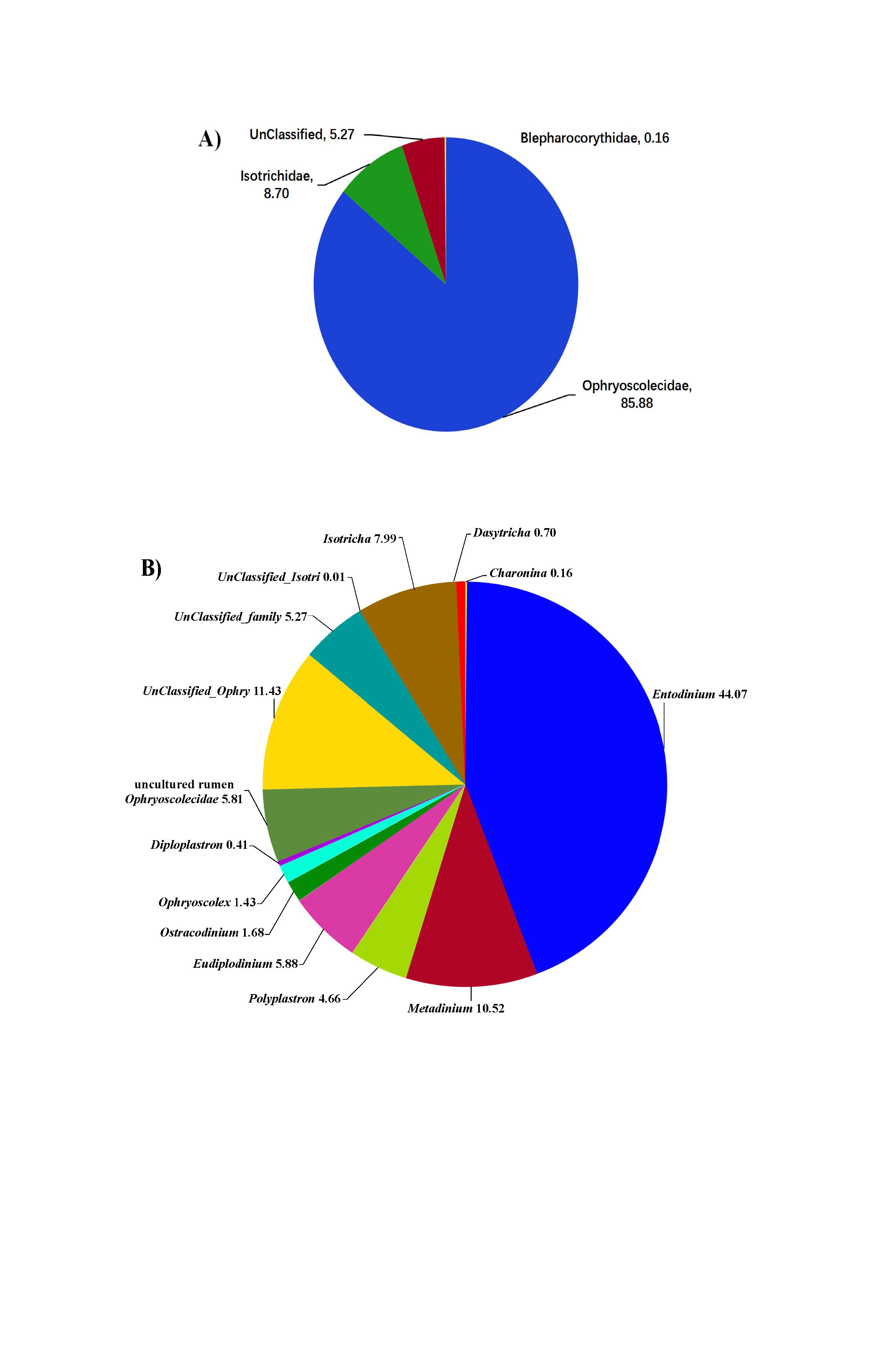

Supplement: Supplementary file 3 — Additional file 3 Figure S1. Relative abundance of ruminal protozoa genera of steers at the family level (A) and the genus level (B). [file 40104_2020_491_MOESM3_ESM.tif]

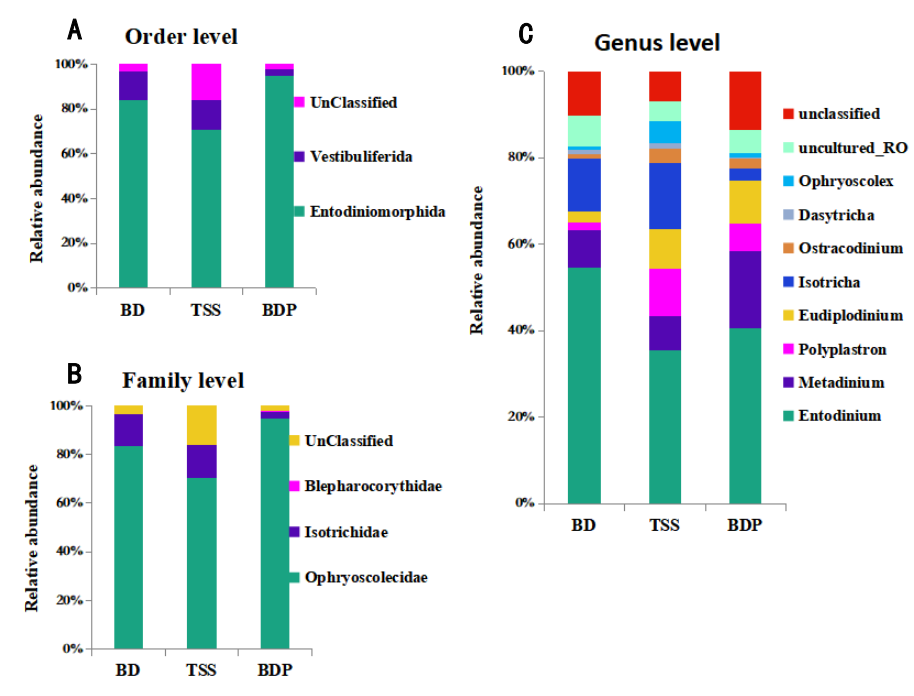

Supplement: Supplementary file 4 — Additional file 3 Figure S2. The community composition of rumen protozoa at the order (A), family (B) and genus (C) level among three periods. [file 40104_2020_491_MOESM4_ESM.png]
